# The protective effect of licochalcone A against inflammation injury of primary dairy cow claw dermal cells induced by lipopolysaccharide

**DOI:** 10.1038/s41598-022-05653-6

**Published:** 2022-01-31

**Authors:** Mengyue Tian, Nan Li, Ruonan Liu, Ke Li, Jinliang Du, Dongmin Zou, Yuzhong Ma

**Affiliations:** 1 Hebei Agricultural University College of Veterinary Medicine, 2596 Lekai South Street, Baoding, 071001 Hebei China; 2 Hebei Agricultural University College of Animal Science and Technology, Baoding, 071001 Hebei China; 3 International Joint Research Laboratory for Fish Immunopharmacology, Freshwater Fisheries Research Center, Chinese Academy of Fishery Sciences, Wuxi, 214081 Jiangsu China; 4 Shanxi Agricultural University College of Veterinary Medicine, Taigu, 030801 Shanxi China

**Keywords:** Pharmacology, Inflammation

## Abstract

Laminitis is one of the most important and intractable diseases in dairy cows, which can lead to enormous economic losses. Although many scholars have conducted a large number of studies on laminitis, the therapeutic test of medicinal plants in vitro is really rare. Licochalcone A is proved to possess anti-inflammatory and anti-oxidant properties. But the effect of licochalcone A on LPS-induced inflammatory claw dermal cells has not been discovered yet. In this study, the primary dairy cow claw dermal cells were treated with gradient concentrations of licochalcone A (1, 5, 10 µg/mL) in the presence of 10 µg/mL lipopolysaccharides (LPS). The results indicated that licochalcone A reduced the concentrations of inflammation mediators (TNF-α, IL-1β and IL-6), increased the activity of SOD, reduced the levels of MDA and ROS, downregulated the mRNA expressions of TLR4 and MyD88, suppressed the protein levels of p-IκBα and p-p65, and upregulated the protein expression of PPARγ. In summary, licochalcone A protected dairy cow claw dermal cells against LPS-induced inflammatory response and oxidative stress through the regulation of TLR4/MyD88/NF-κB and PPARγ signaling pathways.

## Introduction

Laminitis, an aseptic, diffuse, serous inflammation of the dermis, is known as one of the major causes of dairy cow lameness^1^. The prevalence of claw diseases is a significant contributor to the economic losses in the dairy industry^2^. At present, there is still no effective method for the prevention and treatment of laminitis. But proper diet can be a good prevention tool. Antibiotic therapy is still the mainstream therapeutic method to avoid secondary bacterial infections of laminitis in China. However, with the growing concerns about food safety and drug resistance, the research for safer and more effective alternative anti-inflammatory herbs becomes an insistent demand.

Currently, animal models are widely used for the study of bovine laminitis^3^, but the cell models are rarely reported. It showed that inflammatory injury was one of the most important pathological events occurring in the early stages of laminitis^3–5^. As a trigger in the development of inflammatory processes, lipopolysaccharides (LPS) had closely relationship with laminitis^6,7^. Elevated LPS concentrations in plasma were detected in both subclinical and chronic laminitis dairy cows^8^, as well as in the high-grain diet model of goats^9^. In vitro, LPS also had negative effects on lamellar tissue integrity^10^. Therefore, stimulating the dairy cow claw dermal cells with LPS to establish inflammatory model may be a novel way for the research of laminitis.

Liquorice, the root and rhizome of *Glycyrrhiza* species, is a frequently used herb to treat various diseases, including arthritis and gastrointestinal problems^11^. Licochalcone A, one of the major flavonoid compounds isolated from the liquorice^12^, is proved to exert multiple beneficial properties, such as anti-inflammation^13^, antioxidation^14^, anticancer^15^, antibacteria and anti-parasitism^16^. Nevertheless, the effect of licochalcone A on LPS-induced inflammatory claw dermal cells has not been discovered yet.

The objective of current research was to explore the underlying protective effect of licochalcone A on LPS-induced inflammatory claw dermal cells of dairy cow and illuminate the potential mechanisms. The hypothesis was that licochalcone A might protect dairy cow claw dermal cells against LPS-induced inflammatory response and oxidative stress through the regulation of TLR4/MyD88/NF-κB and PPARγ signaling pathways.

## Materials and methods

### Ethics declarations

Claw laminae tissues were collected at a local abattoir from healthy adult dairy cows at Baoding Lianchi slaughterhouse (Hebei, China) and used with the consent of the slaughterhouse. All dairy cows were slaughtered for meat production and no animal was slaughtered specifically for the purpose of tissue collection.

### Reagents

LPS (*Escherichia coli* O55: B5; Cat No. L2630) was purchased from Sigma (St. Louis, USA). Licochalcone A (≥ 98% purity; Cat No. IL0110), dimethyl sulfoxide (DMSO; Cat No. D8371), HE staining kit (Cat No. G1120), Cell counting kit-8 (CCK-8; Cat No.CA1210), RIPA cell lysis buffer (Cat No. R0010), BCA protein assay kit (Cat No. PC0020), NBT/BCIP chromogen kit (Cat No. PR1100) were acquired from Solarbio (Beijing, China). Dulbecco’s Modified Eagle Medium (DMEM; Cat No. 12430-047), fetal bovine serum (FBS; Cat No. 10091-155), Insulin-transferrin-selenium (ITS; Cat No. 41400-045) were purchased from Gibco (Grand Island, NY). ELISA kits of dairy cow tumor necrosis factor-α (TNF-α; Cat No. DG90837Q), interleukin (IL)-1β (Cat No. DG90995Q) and IL-6 (Cat No. DG90838Q) were purchased from DG Biotech Co. Ltd. (Beijing, China). The kits for detection of reactive oxygen species (ROS; Cat No. E004-1-1), malondiadehyde (MDA; Cat No. A003-1-2) and superoxide dismutase (SOD; Cat No. A001-3-2) were purchased from Nanjing Jiancheng Bioengineering Institute (Nanjing, China). Antibodies against vimentin (Cat No. bs-8533R), phosphor-IκBα (Cat No. bsm-52169R), phosphor-p65 NF-κB (Cat No. bs-0982R), p65 NF-κB (Cat No. bs-0465R), PPARγ (Cat No. bs-0530R), and β-actin (Cat No. bs-0061R) were obtained from Bioss (Woburn, MA, USA). HRP-conjugated secondary antibody (Cat No. ZB2308) was acquired from ZSGB-Bio (Beijing, China). Ultrapure RNA extraction kit (Cat No. CW0581M) and Hifiscript cDNA Synthesis Kit (Cat No. CW2569M) were purchased from CWBIO (Beijing, China). 2× Fast Super EvaGreen^®^ qPCR Mastermix (Cat No. S2008) was acquired from US Everbright Inc. (CA, USA).

### Cell isolation and culture

Claw laminae tissues were collected at a local abattoir from adult dairy cows without any visual disease in claw. Dairy cow claw dermal cells were isolated using the tissue adherent culture method as previously described^17^. After shaved, cleansed and washed with 75% ethanol, laminae tissues were collected and put into sterile saline solution with antibiotics (200 units/mL of *Penicillin*, 200 μg/mL of *Streptomycin*). The collected tissues were washed with sterile phosphate buffered saline (PBS), trimmed into small pieces and soaked in 0.25% trypsin solution at 4 °C for 18–24 h. The dermis pieces were seeded in 6-well plates. DMEM supplemented with 15% FBS, 1× ITS, 0.025 M HEPES, 200 units/mL of *Penicillin*, 200 μg/mL of *Streptomycin* was used as cultivation medium. Cells were maintained in 5% CO_2_ at 37 °C. Medium was changed every 2–3 days. Tissue pieces were removed when cells were about 50% confluence. Upon 80–90% confluence, cells were detached with 0.25% trypsin–EDTA solution and seeded into 25 cm^2^ flasks. Cell amount was estimated using hematocytometer (Cat No. YA0810, Solarbio).

For immunofluorescent staining, cells were cultured onto coverslips until 60–70% confluence, fixed with 4% paraformaldehyde solution, and permeated with 0.2% Triton X-100 at room temperature. Afterwards, the cells were blocked with 1% bovine serum albumin (BSA), incubated with anti-vimentin antibody (1:100 dilution) at 37 °C for 2 h, and further incubated with FITC-conjugated secondary antibody (1:100 dilution) at 37 °C for 30 min in the dark. After washed and counterstained with DAPI staining buffer for 5 min in the dark, the cells were observed under an Eclipse E400 fluorescence microscope (Nikon, Vienna, Austria).

For morphological staining, the cells were seeded on cover glass and stained by the HE staining kit, according to the manufacturer's instructions.

### Cell treatment

LPS and licochalcone A were dissolved in PBS (1 mg/mL) and DMSO (50 mg/mL) respectively, and filtered with 0.22 μm microfilter. Then the working solutions of LPS and licochalcone A were diluted with DMEM to final concentrations. The final concentration of DMSO in solution was less than 0.1%.

For viability assay, the primary dermal cells were exposed to gradient concentrations of LPS (1, 5, 10, 50, 100 µg/mL) for 6 h, 12 h, 24 h, 48 h, or licochalcone A (1, 5, 10, 20, 50 µg/mL) for 24 h, 48 h. For other experiments, cells were pretreated with 10 µg/mL LPS for 24 h, and then exposed to 1, 5, 10 µg/mL licochalcone A for 12 h, 24 h, 48 h.

### Cell viability assay

Cell viability was measured using the CCK-8 kit. Dermal cells were seeded into 96-well plates at a density of 5 × 10^4^ cells/well and cultured until 80–90% confluence. After different treatments as mentioned above, 10 µL CCK-8 was added into each well. Put the cell culture plates back into CO_2_ incubator for 1 h. The cell viability was measured by a microplate reader under the wavelength of 450 nm (Bio-Rad, CA, USA).

### Cytokine measurement by ELISA

Dermal cells were seeded into 24-well plates at a density of 2 × 10^5^ cells/well. After different treatment, the concentrations of inflammatory cytokines TNF-α, IL-1β and IL-6 in supernatants were detected using ELISA kits for dairy cow, according to the manufacturer’s guidelines. Calculate the concentrations of cytokines on the basis of standard curve.

### Oxidative stress assay

Dermal cells were seeded into 24-well plates at a density of 2 × 10^5^ cells/well. After different treatment, the level of ROS was detected using the fluorescent oxidant sensing probe 2, 7-dichlorodi-hydrofluorescein diacetate (DCFH-DA) according to the manufacturer’s guideline. After treated as mentioned above, the dermal cells were incubated with 10 μM DCFH-DA at 37 °C for 30 min. The fluorescence was measured using a fluorescent microplate reader (Bio-Rad, CA, USA) at 485 nm excitation and 525 nm emission wavelengths.

The levels of MDA and SOD in supernatants were detected spectrophotometrically following the manufacturer’s protocols. MDA level was measured using the thiobarbituric acid (TBA) method. TBA reacted with MDA to form a pink complex with a peak absorbance at 532 nm, the result was expressed as MDA equivalents (nmol/mg protein). The activity of SOD was measured based on its ability to inhibit the oxidation of oxyamine by the xanthine–xanthine oxidase system. A unit of the enzyme was defined as the amount of enzyme that inhibited the reaction by 50%.

### Western blot analysis

Dermal cells were seeded into 24-well plates at a density of 2 × 10^5^ cells/well. After different treatment, total proteins from dermal cells were extracted with RIPA cell lysis buffer and quantified by BCA protein assay kit. Equivalent protein was denatured, resolved by 12% SDS–polyacrylamide gel, and transferred onto nitrocellulose membranes. Block the nonspecific binding sites of membranes with 5% skimmed milk. Then membranes were hybridized with antibodies specific for phosphor-IκBα, phosphor-p65 NF-κB, p65 NF-κB, PPARγ and β-actin (1:1000 dilution) at 4 °C overnight, following with the HRP-conjugated secondary antibody (1:1000 dilution) incubation for 1 h. Immunoblot signals were visualized with NBT/BCIP chromogen kit. Densitometric values were acquired from 3 independent experiments with the use of Image J software (NIH, Bethesda, MD).

### Quantitative real-time PCR analysis

Dermal cells were seeded into 6-well plates at a density of 1 × 10^6^ cells/well. After different treatment, total RNA was extracted from dermal cells using Ultrapure RNA extraction kit in accordance with the guidelines of manufacturers. The concentration and purity (OD260/OD280 absorption ratio > 1.8) of total RNA were evaluated by a NanoDrop 2000 spectrophotometer (Thermo Scientific, Ottawa, ON, Canada). Subsequently, 2 μg RNA was reverse transcribed into cDNA using Hifiscript cDNA Synthesis Kit following the protocol of manufacturers. Quantitative real-time PCR was accomplished using 2× Fast Super EvaGreen^®^ qPCR Mastermix on a Light Cycler 96 Real-Time PCR system (Roche, Basel, Switzerland). The amplified cycling conditions were carried out as previously described^17^. The 2^−ΔΔCt^ method was used for the calculation of gene expression^18^. The mRNA expression of target genes was normalized by GAPDH. Primers were: toll-like receptor 4 (TLR4), 5′-AGCTTCAACCGTATCATGGCCTCT-3′ (forward), and 5′-ACTAAGCACTGGCATGTCCTCCAT-3′ (reverse); myeloid differentiation factor 88 (MyD88), 5′-AAGTACAAGCCAATGAAGAAAGAG-3′ (forward), and 5′-GAGGCGAGTCCAGAACCAG-3′ (reverse); GADPH, 5′-CACCCTCAAGATTGTCAGCA-3′ (forward), and 5′-GGTCATAAGTCCCTCCACGA-3′ (reverse).

### Statistical analysis

All data were exhibited as mean ± standard error of the mean (SEM) of at least 3 independent experiments. Evaluate the significant differences using one-way analysis of variance (ANOVA), followed by Duncan’s post hoc test using SPSS 21.0 software (SPSS Inc., Chicago, IL). The level of statistically significance was declared as *P* < 0.05.

## Results

### Cell growth and morphologic observation

**Figure 1:**
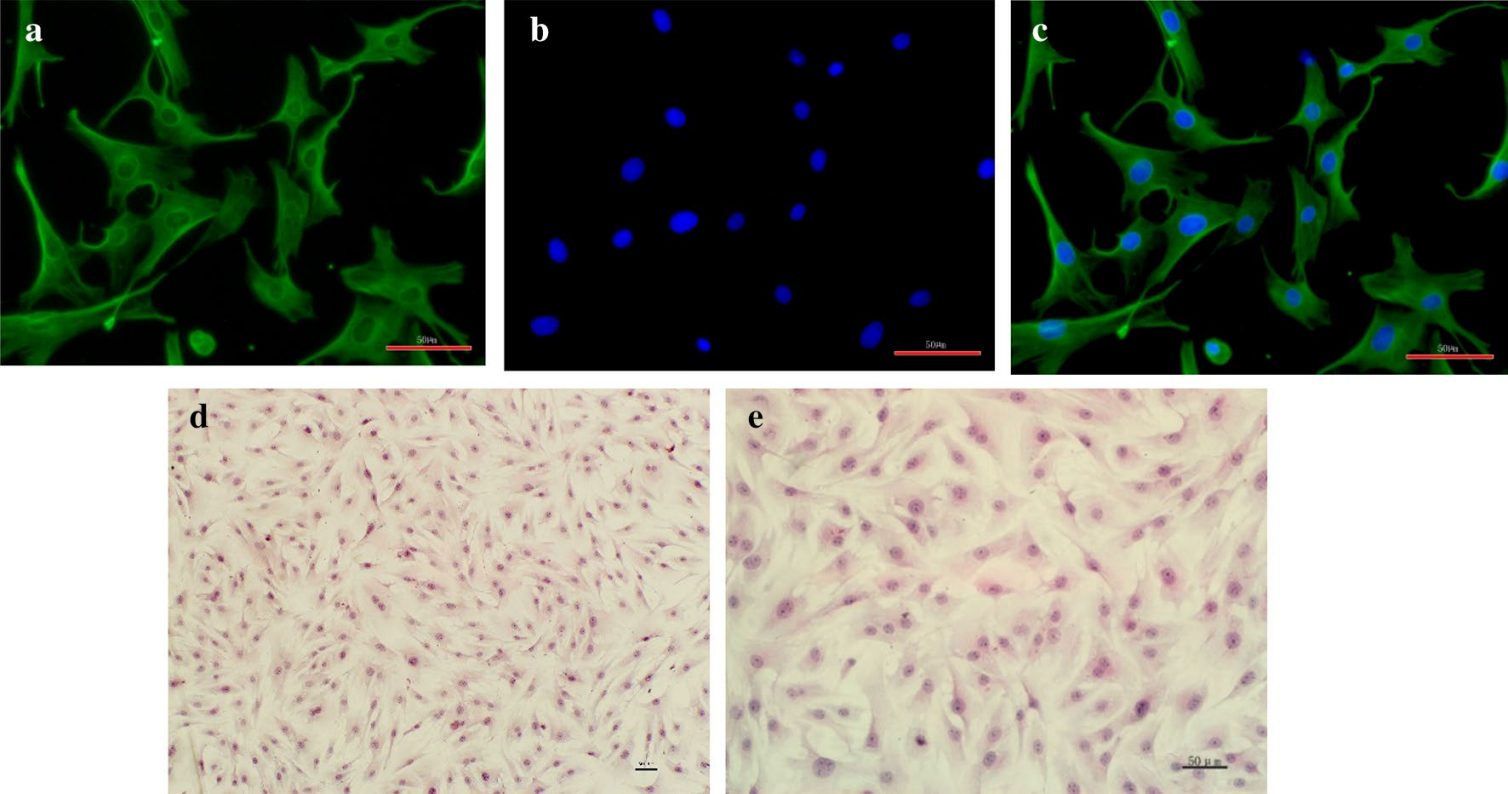
Morphology of primary isolated dairy cow claw dermal cells. (**A**) Cytoplasm was detected of vimentin expression (green); (**B**) Nucleus was stained with DAPI (blue); (**C**) Merge A and B; (**D**,**E**) HE staining. (scale = 50 μm).

Dermal cells started to proliferate from the border of explants after inoculation for 5–7 days. About 2 weeks later, cells reached to 80–90% confluence. As shown in Fig. 1, the dermal cells were irregular in shape, polygonal or long fusiform.

### LPS-induced inflammation injury on claw dermal cells

**Figure 2:**
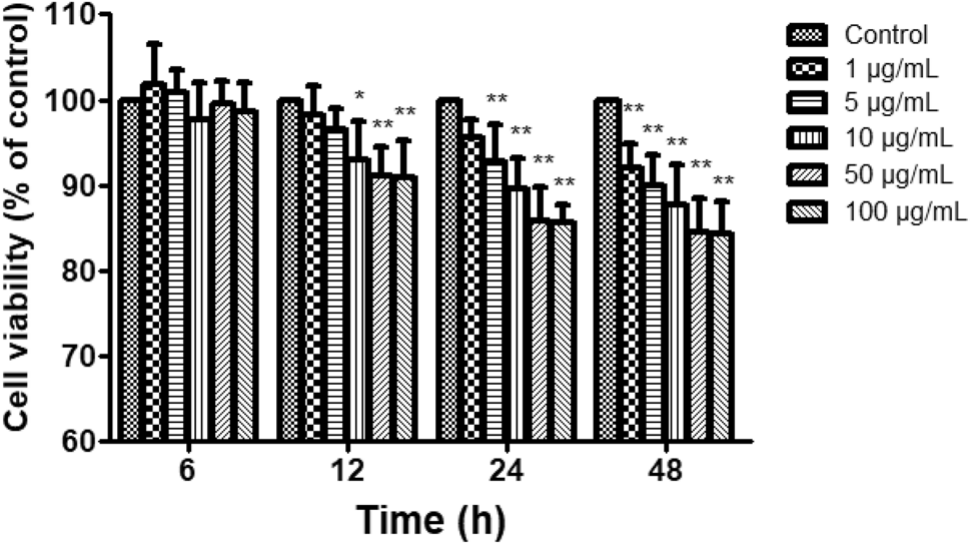
Effect of LPS on the viability of claw dermal cells. Cell viability was measured by cell counting kit-8 (CCK-8) method with the treatment of various concentrations (1, 5, 10, 50, 100 μg/mL) of LPS for 6, 12, 24, 48 h. The data were presented as mean ± SEM (n = 6). *P < 0.05 vs. control group; **P < 0.01 vs. control group.

With 1, 5, 10, 50, 100 μg/mL treatments of LPS, the viability of dermal cells was measured with CCK-8 method to determine the optimal condition for inflammatory model. As shown in Fig. 2, the cell viability had no effect for 6 h treatment of LPS (*P* > 0.05). After 12 h, the inhibition rates of cell viability were significant with 10 μg/mL (*P* < 0.05), 50, 100 μg/mL (*P* < 0.01) LPS treatments, respectively. Cell viabilities were inhibited considerably (*P* < 0.01) with 5, 10, 50, 100 μg/mL LPS treatments for 24 h, and all concentrations of LPS for 48 h. Based on the results, 10 μg/mL LPS was optimal for further research.

### Licochalcone A on the viability of claw dermal cells

**Figure 3:**
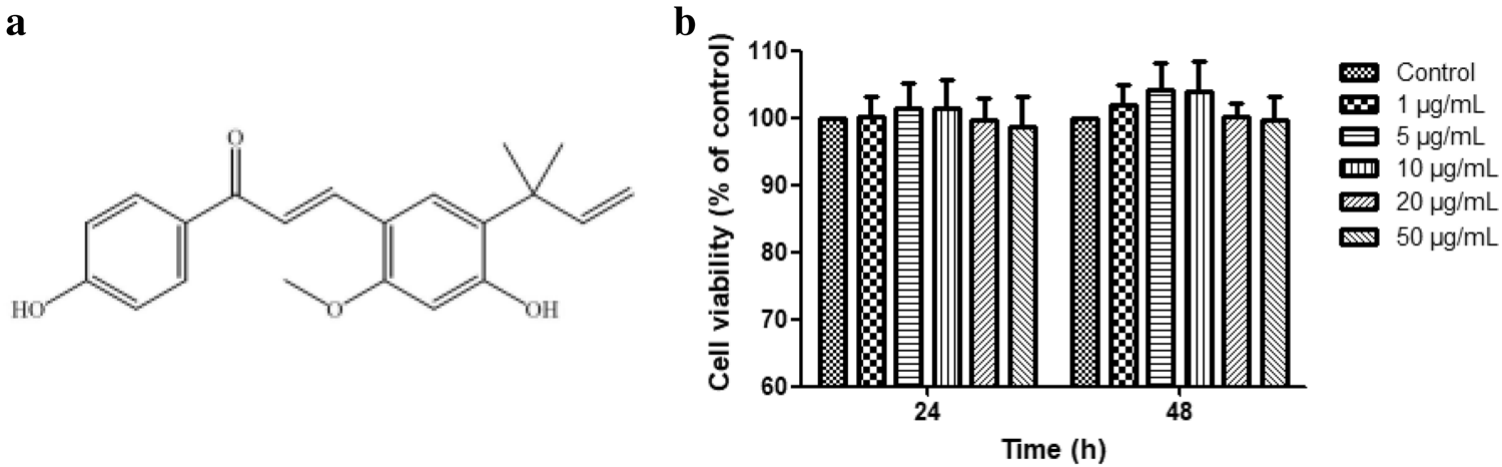
Cytotoxic activity of licochalcone A treatment for 24 h and 48 h on claw dermal cells. (**a**) The chemical structure of licochalcone A. (**b**) Cytotoxic effect was measured by cell counting kit-8 (CCK-8) method with the treatment of various concentrations (1, 5, 10, 20, 50 μg/mL) of licochalcone A for 24 h or 48 h. The data were presented as mean ± SEM (n = 6).

As shown in Fig. 3, the indicated concentrations of licochalcone A treated for 24 h or 48 h had no cytotoxic effects on claw dermal cells (*P* > 0.05). The cell viability was tended to decrease at the concentrations above 20 μg/mL compared with control. Thus, 1, 5, 10 μg/mL licochalcone A were selected as working concentrations for further research.

### Effect of licochalcone A on inflammatory mediators’ secretion

**Figure 4:**
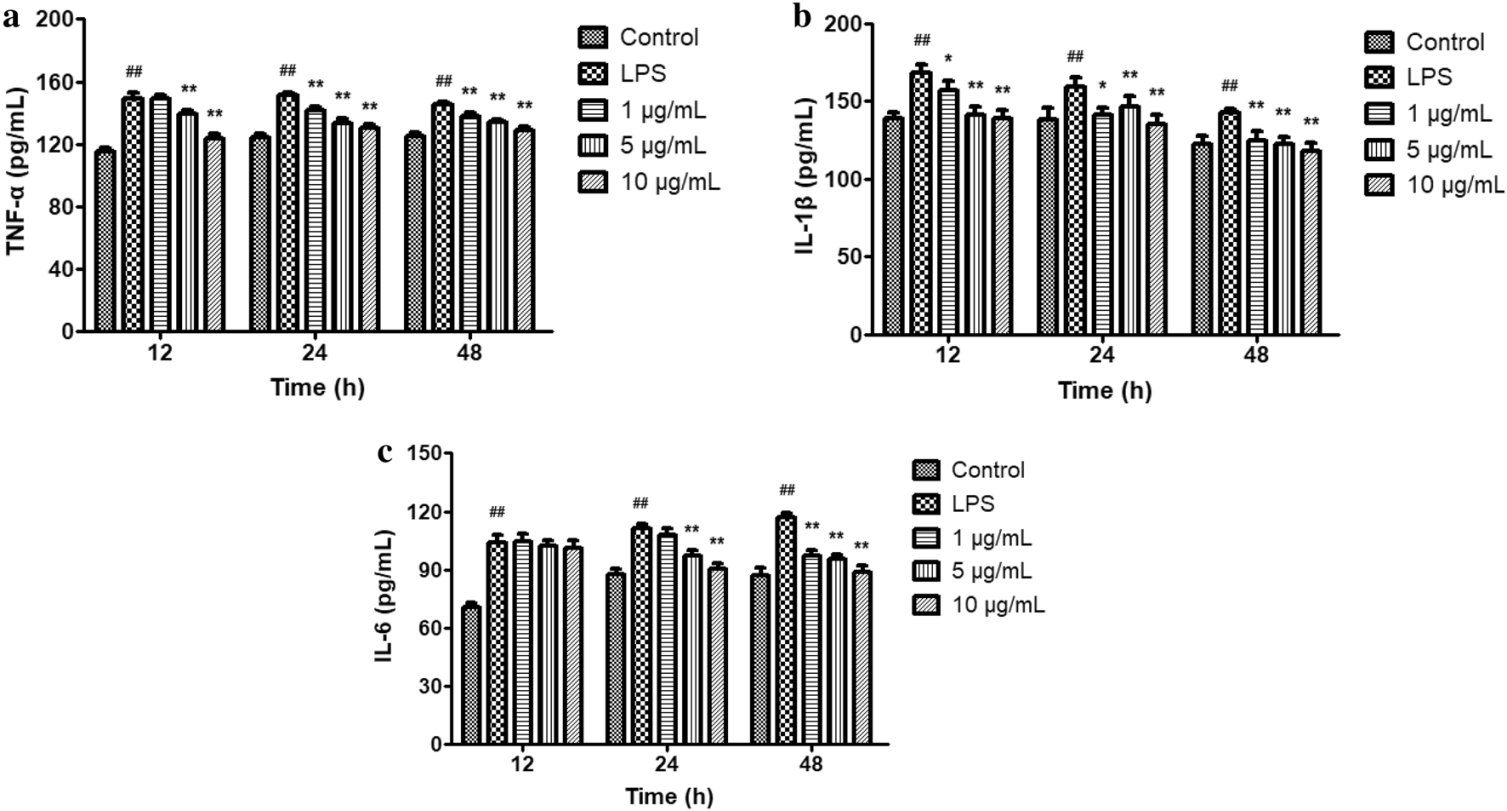
Effect of licochalcone A on LPS-induced secretions of tumor necrosis factor-α (TNF-α) (**a**), interleukin-1β (IL-1β) (**b**) and interleukin-6 (IL-6) (**c**) in claw dermal cells. Cells were exposed to gradient concentrations of licochalcone A (1, 5, 10 μg/mL) with or without the presence of 10 μg/mL LPS for 12, 24, and 48 h, respectively. The data were presented as mean ± SEM (n = 6). ^##^*P* < 0.01 vs. control group; *P < 0.05 vs. LPS model group; **P < 0.01 vs. LPS model group.

It showed that the concentrations of TNF-α, IL-1β as well as IL-6 were higher responding to LPS stimulation (*P* < 0.01) in Fig. 4. Compared with the LPS-treated group, 5, 10 μg/mL licochalcone A significantly reduced the levels of TNF-α for 12 h, while 1, 5, 10 μg/mL licochalcone A significantly reduced the levels of TNF-α for 24 h and 48 h. The levels of IL-1β were notably reduced by 1, 5, 10 μg/mL licochalcone A. The IL-6 secretion decreased for 24 h and 48 h treatment of licochalcone A, whereas there was no difference at 12 h (*P* > 0.05).

### Effect of licochalcone A on oxidative stress

**Figure 5:**
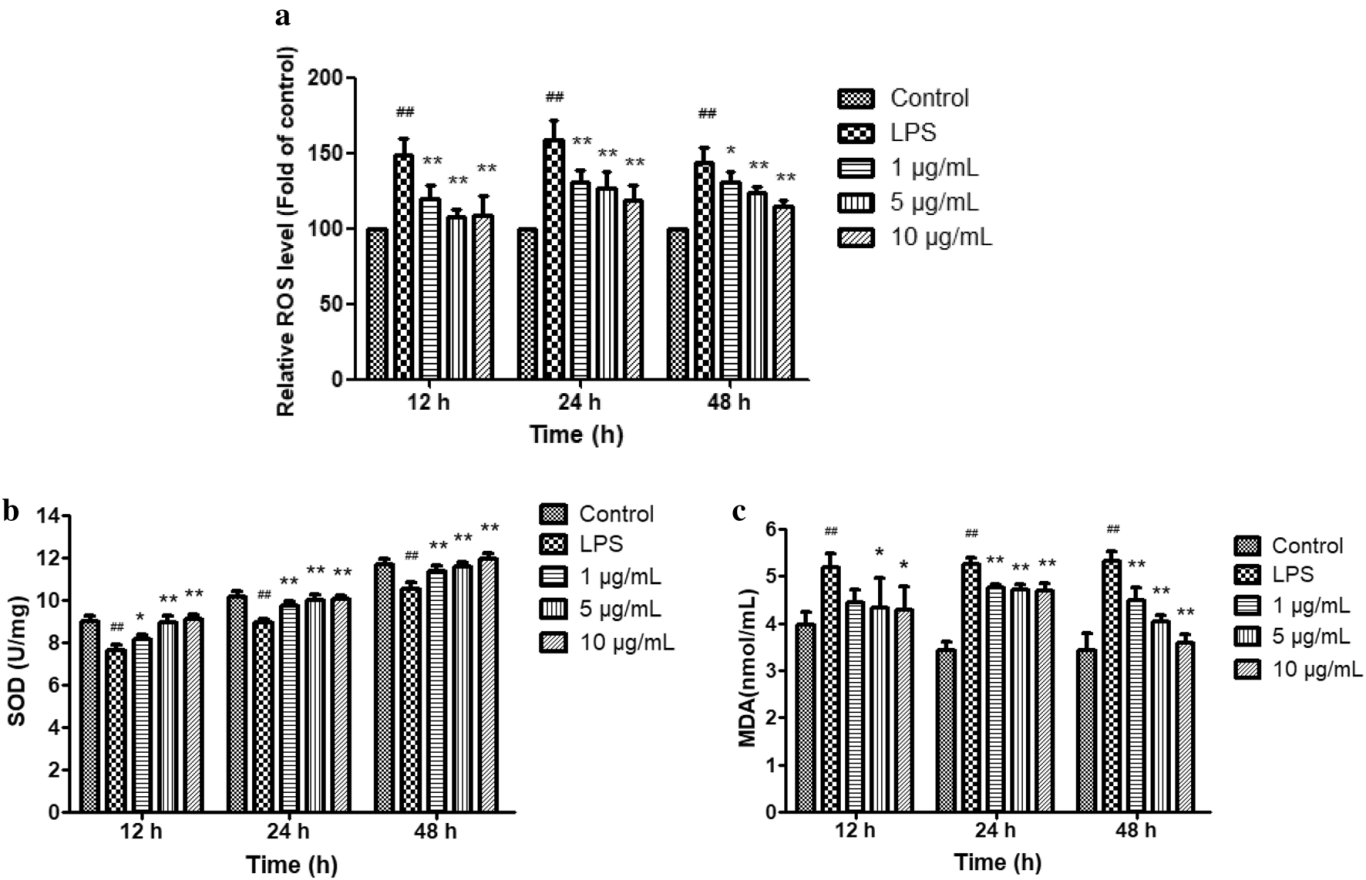
Effect of licochalcone A on LPS-induced levels of reactive oxygen species (ROS) (**a**), superoxide dismutase (SOD) (**b**) and malondiadehyde (MDA) (**c**) in claw dermal cells. Cells were exposed to gradient concentrations of licochalcone A (1, 5, 10 μg/mL) with or without the presence of 10 μg/mL LPS for 12, 24, and 48 h. The data were presented as mean ± SEM (n = 6). ^##^*P* < 0.01 vs. control group; *P < 0.05 vs. LPS model group; **P < 0.01 vs. LPS model group.

As lipid peroxidation indicators, the levels of ROS, SOD and MDA were detected to assess the antioxidative capacity of licochalcone A on LPS-induced inflammatory dermal cells. In comparison with the control group, the activity of SOD reduced and the levels of ROS and MDA increased after LPS treatment (*P* < 0.01) (Fig. 5). In response to licochalcone A treatment, the ROS level reduced significantly (*P* < 0.01). The SOD level significantly enhanced (*P* < 0.01) time-dependently and dose-dependently. Licochalcone A treatment significantly reduced the MDA content, except 1 μg/mL treatment for 12 h (Fig. 5).

### Effect of licochalcone A on protein expression of PPARγ, p-IκB and p-p65

**Figure 6:**
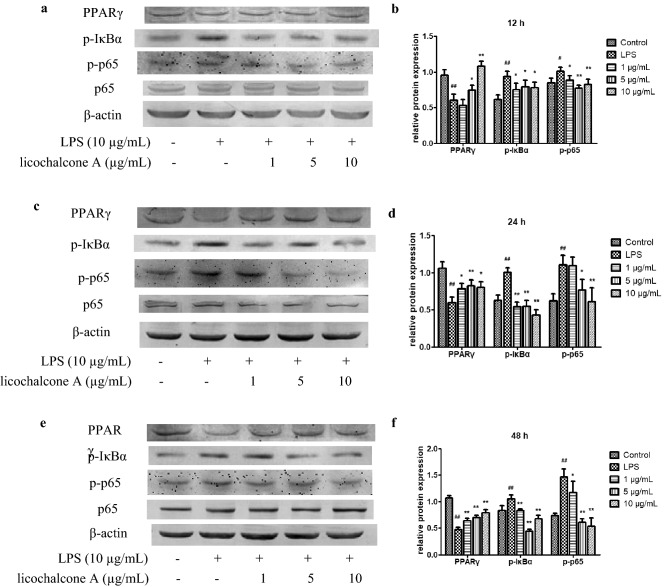
Effect of licochalcone A on LPS-induced protein levels of peroxisome proliferator-activated receptor γ (PPARγ), p-κB inhibitor α (IκBα) and p-p65 in claw dermal cells after treatment for 12 h (**a**,**b**), 24 h (**c**,**d**) and 48 h (**e**,**f**). Cells were exposed to gradient concentrations of licochalcone A (1, 5, 10 μg/mL) with or without the presence of 10 μg/mL LPS for 12, 24, and 48 h, respectively. The relative protein expressions of PPARγ/β-actin, p-IκBα/β-actin and p-p65/p65 were measured. The data were presented as mean ± SEM (n = 6). ^##^*P* < 0.01 vs. control group; ^#^*P* < 0.05 vs. control group; *P < 0.05 vs. LPS model group; **P < 0.01 vs. LPS model group (images are cropped. The full-length blots are presented in Supplementary file).

As exhibited in Fig. 6, the protein expression of PPARγ decreased in response to LPS challenge (*P* < 0.01). The expression of PPARγ increased significantly with 1 μg/mL licochalcone A treatment for 24 h and 48 h, and 5, 10 μg/mL treatments for 12 h, 24 h and 48 h, respectively.

Additionally, the total protein expressions of p-p65 and p-IκBα enhanced (*P* < 0.01) with LPS treatments. Compared with LPS treated group, the phosphorylation of IκBα obviously reduced with 1, 5, 10 μg/mL licochalcone A treatments for 12 h, 24 h and 48 h, respectively. The phosphorylation of p65 decreased with 1 μg/mL licochalcone A treatment for 12 h and 48 h, as well as 5, 10 μg/mL treatment for 12 h, 24 h and 48 h.

### Effect of licochalcone A on mRNA expressions of TLR4 and MyD88

**Figure 7:**
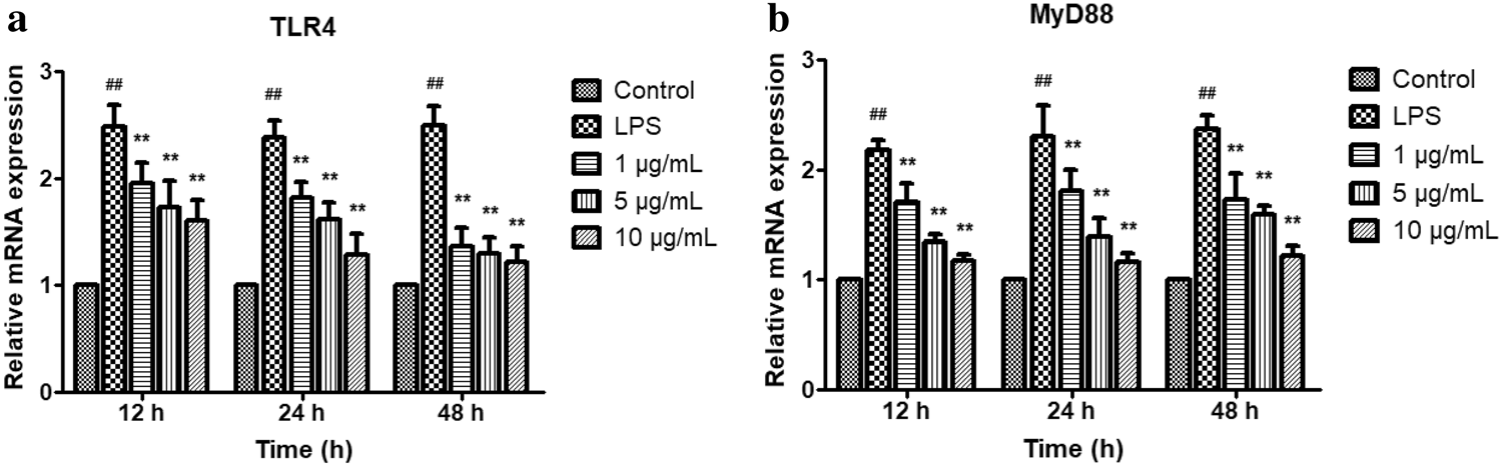
Effect of licochalcone A on LPS-induced mRNA expressions of toll-like receptor 4 (TLR4) (**a**) and myeloid differentiation factor 88 (MyD88) (**b**) in dermal cells. Cells were exposed to gradient concentrations of licochalcone A (1, 5, 10 μg/mL) with or without the presence of 10 μg/mL LPS for 12, 24, and 48 h respectively. The gene expressions of TLR4 and MyD88 were quantified using PCR analysis. The data were presented as mean ± SEM (n = 6). ^##^*P* < 0.01 vs. control group; *P < 0.05 vs. LPS model group; **P < 0.01 vs. LPS model group.

LPS markedly upregulated the mRNA expressions of TLR4 and MyD88 (*P* < 0.01), these gene expressions were suppressed significantly by licochalcone A treatment for 12 h, 24 h and 48 h in a dose-dependent manner (*P* < 0.01) (Fig. 7).

## Discussion

In the current study, it demonstrated that licochalcone A was able to attenuate the inflammation response caused by LPS on claw dermal cells with the regulation of TLR4/MyD88/NF-κB pathway and PPARγ expression, which provided a new idea for the possibility of licochalcone A being used for the treatment of bovine laminitis.

Dairy cow laminitis, an aseptic inflammation of the claw dermal layer, is one of the most prevalent diseases, which seriously impacts the dairy cow welfare and the development of dairy industry^19^. The prevention and treatment of laminitis depends on claw trimming and the improvement of feeding management measures. Nutrition is also essential for the immunoregulation and inflammation treatment of animals^20^. In recent years, medicinal plants have been studied increasingly as alternative or additional drugs due to their low drug resistance and multi-target therapeutic properties^21^. It provides a new idea for the possibility of medicinal plants being used for the treatment of laminitis. In current research, LPS was used as an initiator for inflammation injury model to explore the possible anti-inflammatory effects of licochalcone A on dairy cow claw dermal cells.

A rapid inflammatory response stimulated by LPS is characterized by the overproduction of inflammatory mediators^22^. In our results, it demonstrated that LPS inhibited the cell viability and markedly enhanced the secretion of TNF-α, IL-1β and IL-6, indicating that the inflammation model of dermal cells was induced successfully. Inflammatory cytokines’ reduction could serve as a vital therapy signal for the inflammation amelioration. Licochalcone A restrained effectively the secretions of TNF-α, IL-1β as well as IL-6, thus exerted its anti-inflammatory capacity as previous studies showed^23^.

Inflammation and oxidative stress are two closely relative pathological processes, which could be modulated through specific signaling pathways like NF-κB and peroxisome proliferator-activated receptor (PPAR)^24,25^. On the one hand, the process of inflammation could produce a number of soluble mediators and activate signal transduction cascades, leading to exaggerated oxidative stress^26^. In addition, oxidative stress could cause biomacromolecule damage and induce inflammatory response via the initiation of various signaling pathways^27^. Inflammation stimulated the cellular production of ROS, meanwhile the excessive ROS enhanced the inflammatory response in turn^24^. As classic indicators of lipid peroxidation, SOD was a pivotal antioxidant enzyme for superoxide scavenging, and MDA was a catabolite of fatty acid oxidation^28^. In this research, it demonstrated that the treatment of licochalcone A was capable of attenuating the oxidative damage during the LPS-induced inflammation injury, evidenced by enhancement of the SOD activity as well as the reduction of the ROS and MDA content.

Next, we investigated the potential mechanisms underlying the licochalcone A -mediated protection against LPS-induced injury. It is well known that TLR4 is of prime importance to the inflammation signal caused by LPS^29^. Via stimulation of MyD88, the TLR4-LPS complex is capable of initiating a series of cascade reactions, results in the degradation of κB inhibitor α (IκBα), the activation of nuclear factor-κB (NF-κB), and eventually regulates the secretion of inflammatory mediators^30,31^. In this paper, we investigated the relationship between the inhibition of licochalcone A on inflammatory indicators secretion and the regulation of TLR4/MyD88 signaling pathway. As expected, the mRNA levels of TLR4 and MyD88 on dairy cow claw dermal cells were upregulated by LPS. This effect was suppressed dose-dependently by licochalcone A, which protected dermal cells from inflammation injury.

Previous research has confirmed that PPARγ possessed potent effects of anti-inflammatory and antioxidative^32,33^. Thus, we speculated that PPARγ related to the protective effects of licochalcone A against LPS-induced inflammatory dermal cells. Moreover, PPARs were referred to be one of the therapeutic targets for insulin resistance laminitis in the horse^34^. In this paper, the role of PPARγ in the inflammation response of dairy cow dermal cells was explored. The results showed that licochalcone A might exert its protective effect from LPS-induced inflammatory injury through enhancing PPARγ expression.

NF-κB is recognized as a crucial inflammatory mediator, heterodimer p50-p65 is its common form^35,36^. In an unstimulated state, NF-κB presents an inactivated state because of the binding with IκB, an inhibitory protein. Activators like LPS could trigger the phosphorylation, ubiquitination, and proteasomal degradation of IκB proteins via TLR4/MyD88 signaling pathway, resulting in the separation of NF-κB/IκB complex. Afterwards, the NF-κB dimers dissociated from IκB, transferred into nucleus, and participated in the transcription and expression of inflammatory cytokines^37^. Thus, the suppression of NF-κB activation serves as a key mechanism for amelioration of inflammatory injury^38^. In this data, it showed that licochalcone A obviously restrained the initiation of NF-κB pathway by decreasing the phosphorylation of IκBα and p65, resulting in the amelioration of inflammation injury induced by LPS.

However, there are still some shortcomings in our study. The limitations of in vitro cell model experiment do not allow an exact replication of the pathological process of bovine laminitis. In the future, a series of in vivo experiments will be performed to test the anti-inflammatory effect of licochalcone A on bovine laminitis.

In conclusion, our results demonstrate that licochalcone A protects dairy cow claw dermal cells against LPS-induced inflammatory injury and oxidative stress through the regulation of PPARγ and TLR4/MyD88/NF‐κB signaling pathways, thereby reducing the secretion of TNF‐α, IL‐1β and IL‐6, and regulating the levels of ROS, SOD and MDA.

## Supplementary Information

Supplementary Information.

## Data Availability

The datasets used and/or analyzed during the current study are available from the corresponding author on reasonable request.
