# Supplementary material for: The protective effect of licochalcone A against inflammation injury of primary dairy cow claw dermal cells induced by lipopolysaccharide
Source: Sci Rep. 2022 Jan 31;12:1593. doi: 10.1038/s41598-022-05653-6 (PMC8803976; doi:10.1038/s41598-022-05653-6)
Supplement: Supplementary file 1 — Supplementary Information. [file 41598_2022_5653_MOESM1_ESM.docx]

Supplementary file 1. The original, full length blots of western blot.

a. 12 h


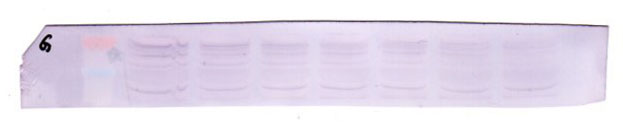


PPARγ

57 kDa

80 kDa

60 kDa

LPS (10 µg/mL) - + + + +

licochalcone A (µg/mL) - - 1 5 10


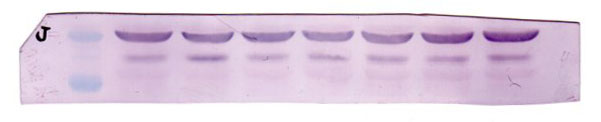


p-IĸBα

LPS (10 µg/mL) - + + + +

licochalcone A (µg/mL) - - 1 5 10

35 kDa

45 kDa

36 kDa


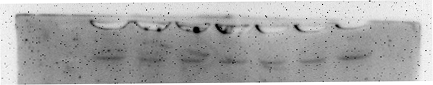


p-p65

60 kDa

61 kDa

LPS (10 µg/mL) - + + + +

licochalcone A (µg/mL) - - 1 5 10


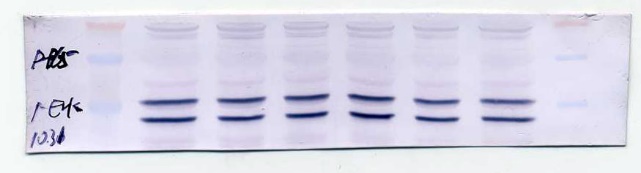


p65

61 kDa

60 kDa

80 kDa

LPS (10 µg/mL) - + + + +

licochalcone A (µg/mL) - - 1 5 10


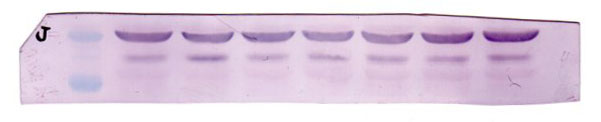


β-actin

45 kDa

42 kDa

35 kDa

LPS (10 µg/mL) - + + + +

licochalcone A (µg/mL) - - 1 5 10

b. 24 h


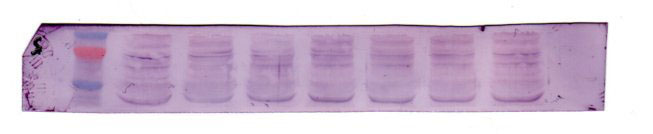


PPARγ

57 kDa

80 kDa

60 kDa

LPS (10 µg/mL) - + + + +

licochalcone A (µg/mL) - - 1 5 10


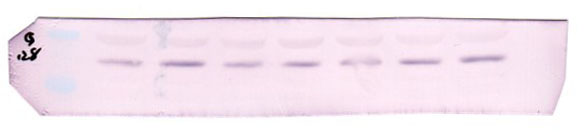


p-IĸBα

LPS (10 µg/mL) - + + + +

licochalcone A (µg/mL) - - 1 5 10

35 kDa

45 kDa

36 kDa


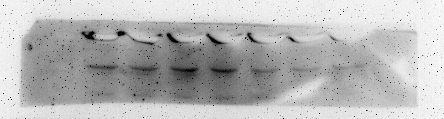


p-p65

60 kDa

61 kDa

LPS (10 µg/mL) - + + + +

licochalcone A (µg/mL) - - 1 5 10


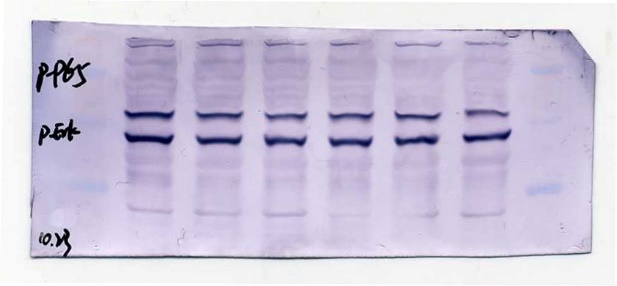


p65

61 kDa

60 kDa

80 kDa

LPS (10 µg/mL) - + + + +

licochalcone A (µg/mL) - - 1 5 10


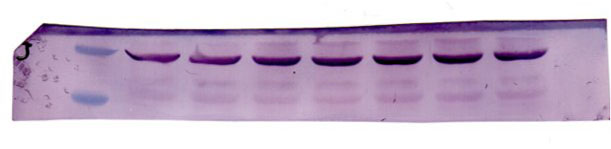


β-actin

45 kDa

42 kDa

35 kDa

LPS (10 µg/mL) - + + + +

licochalcone A (µg/mL) - - 1 5 10

c. 48 h


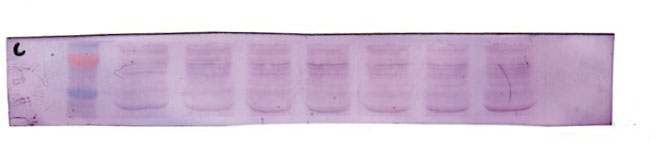


PPARγ

57 kDa

80 kDa

60 kDa

LPS (10 µg/mL) - + + + +

licochalcone A (µg/mL) - - 1 5 10


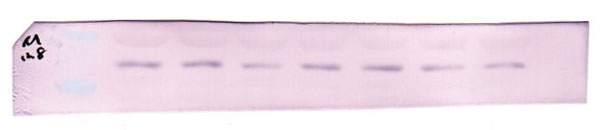


p-IĸBα

LPS (10 µg/mL) - + + + +

licochalcone A (µg/mL) - - 1 5 10

35 kDa

45 kDa

36 kDa


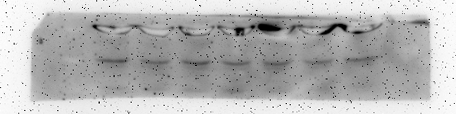


p-p65

60 kDa

61 kDa

LPS (10 µg/mL) - + + + +

licochalcone A (µg/mL) - - 1 5 10


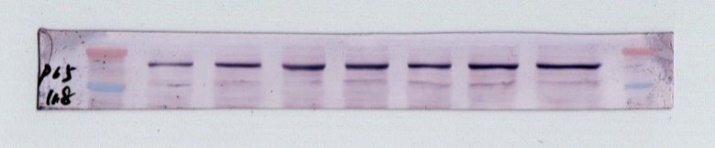


p65

61 kDa

60 kDa

80 kDa

LPS (10 µg/mL) - + + + +

licochalcone A (µg/mL) - - 1 5 10


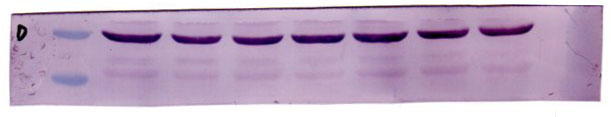


β-actin

45 kDa

42 kDa

35 kDa

LPS (10 µg/mL) - + + + +

licochalcone A (µg/mL) - - 1 5 10
